# Supplementary material for: Protein status of people with phenylketonuria: a scoping review protocol
Source: BMJ Open. 2021 Sep 14;11(9):e049883. doi: 10.1136/bmjopen-2021-049883 (PMC8442069; doi:10.1136/bmjopen-2021-049883)
Supplement: Supplementary data [file bmjopen-2021-049883supp001.pdf]

Supplemental material: Search strategy

Database: MEDLINE (Ovid) < 1946 to May 17, 2021>

Search	Query	Records retrieved
#1	exp Phenylketonurias/ OR Phenylketonurias.mp. OR PKU.mp. OR Hyperphenylalanin?emia.mp. OR exp Phenylalanine Hydroxylase/ OR "Phenylalanine Hydroxylase".mp. OR "Phenylalanine Hydroxylase deficiency".mp.	8935
#2	exp Nutritional Status/ OR "nutritional status".mp. OR "protein status".mp. OR exp Muscle Proteins/ OR "muscle proteins".mp. OR "protein metabolism".mp. OR "muscle protein metabolism".mp. OR exp Body Composition/ OR "body composition".mp. OR exp Muscle Strength/ OR "muscle strength".mp. OR "muscle function".mp. OR exp Prealbumin/ OR prealbumin.mp. OR Transthyretin.mp. OR exp Albumins/ OR albumin.mp. OR "3-methylhistidine concentrations".mp. OR exp Retinol-Binding Proteins/ OR "retinol-binding protein".mp. OR "urea production".mp. OR exp Nitrogen/ OR nitrogen.mp. OR exp Creatinine/ OR creatinine.mp. OR “VO2max” OR exp Physical Exertion/ OR "physical exertion".mp. OR exp Exercise Test/ OR "exercise test".mp.	1093416
#3	#1 AND #2	**282**
Limit	English only	**255**

Firman S, *et al. BMJ Open* 2021; 11:e049883. doi: 10.1136/bmjopen-2021-049883
