# Supplementary material for: Protein status of people with phenylketonuria: a scoping review protocol
Source: BMJ Open. 2021 Sep 14;11(9):e049883. doi: 10.1136/bmjopen-2021-049883 (PMC8442069; doi:10.1136/bmjopen-2021-049883)
Supplement: Author's manuscript [file bmjopen-2021-049883.draft_revisions.pdf]

BMJ Open is committed to open peer review. As part of this commitment we make the peer review history of every article we publish publicly available.

When an article is published we post the peer reviewers' comments and the authors' responses online. We also post the versions of the paper that were used during peer review. These are the versions that the peer review comments apply to.

The versions of the paper that follow are the versions that were submitted during the peer review process. They are not the versions of record or the final published versions. They should not be cited or distributed as the published version of this manuscript.

BMJ Open is an open access journal and the full, final, typeset and author-corrected version of record of the manuscript is available on our site with no access controls, subscription charges or pay-per-view fees (http://bmjopen.bmj.com).

If you have any questions on BMJ Open's open peer review process please email info.bmjopen@bmj.com

Protein status of people with phenylketonuria: A scoping review protocol

Journal:	*BMJ Open*
Manuscript ID	bmjopen-2021-049883
Article Type:	Protocol
Date Submitted by the Author:	07-Feb-2021
Complete List of Authors:	Firman, Sarah; King's College London, Department of Nutritional Sciences; Guy's and St Thomas' NHS Foundation Trust, Adult Inherited Metabolic Diseases<br>Ramachandran, Radha; Guy's and St Thomas' NHS Foundation Trust, Adult Inherited Metabolic Diseases<br>Whelan, Kevin; King's College London, Department of Nutritional Sciences<br>Witard, Oliver ; King's College London, Department of Nutritional Sciences; King's College London, Centre for Human and Applied Physiological Sciences<br>O'Keeffe, Majella; King's College London, Department of Nutritional Sciences; University College Cork, School of Food and Nutritional Sciences
Keywords:	Protocols & guidelines < HEALTH SERVICES ADMINISTRATION & MANAGEMENT, NUTRITION & DIETETICS, GENETICS

SCHOLARONE™
Manuscripts

I, the Submitting Author has the right to grant and does grant on behalf of all authors of the Work (as defined in the below author licence), an exclusive licence and/or a non-exclusive licence for contributions from authors who are: i) UK Crown employees; ii) where BMJ has agreed a CC-BY licence shall apply, and/or iii) in accordance with the terms applicable for US Federal Government officers or employees acting as part of their official duties; on a worldwide, perpetual, irrevocable, royalty-free basis to BMJ Publishing Group Ltd ("BMJ") its licensees and where the relevant Journal is co-owned by BMJ to the co-owners of the Journal, to publish the Work in this journal and any other BMJ products and to exploit all rights, as set out in our licence.

The Submitting Author accepts and understands that any supply made under these terms is made by BMJ to the Submitting Author unless you are acting as an employee on behalf of your employer or a postgraduate student of an affiliated institution which is paying any applicable article publishing charge ("APC") for Open Access articles. Where the Submitting Author wishes to make the Work available on an Open Access basis (and intends to pay the relevant APC), the terms of reuse of such Open Access shall be governed by a Creative Commons licence – details of these licences and which Creative Commons licence will apply to this Work are set out in our licence referred to above.

Other than as permitted in any relevant BMJ Author's Self Archiving Policies, I confirm this Work has not been accepted for publication elsewhere, is not being considered for publication elsewhere and does not duplicate material already published. I confirm all authors consent to publication of this Work and authorise the granting of this licence.

For peer review only

Protein status of people with phenylketonuria: A scoping review protocol

Sarah Firman[1,2,3], Radha Ramachandran[3], Kevin Whelan[1], Oliver C. Witard[1,4], Majella O'Keeffe[1,5]

1. Department of Nutritional Sciences, King's College London, London, United Kingdom
2. Department of Nutrition and Dietetics, Guy's and St Thomas' NHS Foundation Trust, London, United Kingdom
3. Adult Inherited Metabolic Diseases, Guy's and St Thomas' NHS Foundation Trust, London, United Kingdom
4. Centre for Human and Applied Physiological Sciences, King's College London, London, United Kingdom
5. School of Food and Nutritional Sciences, University College Cork, College Road, Cork, Ireland

Corresponding author:

Dr Majella O'Keeffe
School of Food and Nutritional Sciences
Biosciences Institute, Room 4.08
University College Cork
Cork
Ireland
Email: majella.okeeffe@ucc.ie

Word count: 2734 words

For peer review only

ABSTRACT

Introduction: Phenylketonuria (PKU) is a disorder of protein metabolism resulting in an accumulation of phenylalanine in the body. Dietary management consists of altering the sources of ingested protein to limit phenylalanine intake. Current dietary protein guidelines for PKU are based on limited scientific evidence, thus it remains unclear whether current practice leads to optimal protein status in people with PKU. To date, no attempt has been made to systematically evaluate the protein status of people with PKU, using a combination of validated anthropometric, biochemical and functional measurement tools. Furthermore, factors known to influence protein status in the general population warrant consideration when determining protein status in individuals with PKU, alongside factors unique to PKU such as the type of protein substitute consumed. Understanding the impact of these variables on protein status is crucial to developing a personalised approach to protein recommendations for optimising health and functional outcomes in people with PKU. Therefore, the aim of this scoping review is to examine existing evidence regarding the protein status of people with PKU, and to investigate the nutritional and lifestyle variables that influence protein status.

Methods and analysis: This review will be guided by Arksey and O'Malley's framework, along with guidance from Levac et al, Pawliuk et al, and the Joanna Briggs Institute. The following databases will be searched: MEDLINE(Ovid), Embase, CENTRAL, Web of Science and Scopus, alongside grey literature. Identified literature will be assessed by two independent reviewers for inclusion. Descriptive numerical analysis will be performed and a narrative summary will accompany the tabulated results describing how study findings relate to the review questions.

Ethics and dissemination: This review protocol does not require ethical approval. Findings will be disseminated through peer-reviewed publication, presented at relevant conferences, and shared with a patient research advisory group to inform discussions on future research.

Keywords: body composition; muscle function; phenylketonuria; protein blood levels; protein status

For peer review only

For peer review only - http://bmjopen.bmj.com/site/about/guidelines.xhtml

ARTICLE SUMMARY

Strengths and limitations of this study

- This review is the first to collate data from various anthropometric, biochemical and functional measurements of protein status in people with PKU.
- With limited research in the area of PKU, the broad research question will allow for the mapping of available evidence, and to identify the gaps in existing research to inform the direction of further research into improving the care provided for people with PKU.
- The search strategy includes identification of published and unpublished literature, including sourcing grey literature by contacting specialist groups and hand searching conference and meeting reports.
- The protocol includes a clearly defined inclusion criteria aligned with Joanna Briggs Institute PCC strategy to ensure transparency of information sourced for evaluation in the review.
- Aligned with the accepted framework for scoping reviews, this study does not include formal assessment of the quality of evidence.

For peer review only

For peer review only - http://bmjopen.bmj.com/site/about/guidelines.xhtml

INTRODUCTION

Phenylketonuria (PKU; OMIM 261600) is an inborn error of protein metabolism that leads to an accumulation of phenylalanine in the blood and brain. Early diagnosis and treatment are essential in preventing profound and irreversible intellectual disability, in addition to reducing other neurological consequences associated with high phenylalanine throughout life. Lifelong treatment is recommended for individuals with blood phenylalanine levels above the country-specific phenylalanine targets. Currently, dietary management provides the mainstay of treatment and consists of altering the sources of ingested protein to limit phenylalanine intake, and supplementation with phenylalanine-free or low-phenylalanine protein sources to meet protein requirements. In practice, this dietary regimen involves restricting protein intake from food and instead providing the majority (up to 80%) of protein intake from phenylalanine-free or low-phenylalanine protein substitutes, consisting of either L-amino acids (L-AA) or casein glycomacropeptide (CGMP).

A key limitation of existing guidelines on dietary protein requirements for people with PKU[1–3] is that the data has been extrapolated from studies that estimated protein requirements in healthy populations[4–7]. These studies utilised nitrogen balance methodology to evaluate the minimum protein (i.e. nitrogen) intake required to balance nitrogen losses at the whole-body level of physiology[8]. In contrast to the definition of a protein requirement, a protein recommendation serves to optimise metabolic function and improve health and functional outcomes in a given population group[9,10]. Experimental studies conducted in healthy young (18-35 year old) adults without PKU provide useful insight into the impact of L-AA supplementation on plasma amino acid kinetics[11–15]. The rapid rise in plasma amino acid concentrations observed with ingestion of L-AA has been shown to result in greater amino acid oxidation rates and lower protein retention at the whole-body level in comparison with whole protein. To account for the reduced uptake and utilisation of amino acids from protein substitutes, current advice is to apply a correction factor of 20-40% in excess of the protein requirement guidelines of the general population[1–3]. However, the evidence underpinning protein requirement guidelines for dietary management of adults with PKU is limited, and in their current form may not adequately offset age-related changes in protein metabolism across the adult lifespan in people with PKU[7].

In addition to total daily protein intake, multiple factors are known to influence protein status in the non-PKU population. These factors include, but are not limited to, the protein dose ingested per meal/serving, protein source, the timing of protein intake in relation to physical activity and/or other nutrients, the daytime distribution pattern of protein intake, and also the co-ingestion of other nutrients alongside protein[16–22]. Furthermore, age, sex and physical activity status are all known to modify the metabolic fate of ingested protein[21–25]. These variables are also relevant when considering research undertaken to determine protein status in individuals with PKU. In addition, there are factors unique to the dietary regimen of people with PKU that warrant consideration such as type of protein substitute (L-AA versus CGMP), dose and timing of protein substitute ingestion, the proportion of total protein intake from dietary protein versus protein substitutes, adherence to protein substitute prescription, and the period of time spent on and off a phenylalanine restricted diet.

For peer review only - http://bmjopen.bmj.com/site/about/guidelines.xhtml

It remains unclear whether existing protein requirement guidelines for people with PKU translate to optimal protein status across their lifespan. Developing an understanding of the variables that modulate protein status, both positively and negatively, will be crucial to informing future research on personalising protein recommendations for individuals of all ages with PKU, with the intention of improving the care provided for people with PKU. The term ‘protein status’ encompasses a vast range of measurements and physiological outcomes, and for the purpose of this scoping review the term will include those parameters detailed in Table 1. Studies investigating body composition and biochemical measurements of protein nutritional status have been conducted in children and adults with PKU[26–30], yet no contemporary synthesis has been undertaken. Mapping the available evidence is important to identify the direction(s) for future research into establishing evidence-based protein recommendations for dietary management of PKU. In this regard, a scoping review provides the ideal approach to explore the breadth of literature on the protein status of people with PKU in order to map and synthesise the evidence and identify the key variables that influence protein status in this population.

To date, no attempt has been made to systematically evaluate the protein status of people with PKU, using a combination of validated anthropometric, biochemical and functional measurement tools. Although three systematic reviews of protein status in people with PKU are currently registered on PROSPERO, two are exclusively focused on bone health (which is not the focus of the proposed review), with the other systematic review and meta-analysis investigating measurements of body composition only, in patients with PKU[31]. Our scoping review is unique in taking a holistic approach by encompassing a range of validated measurements of protein status, including biochemical (for example 3-methylhistidine concentrations, pre-albumin, and urinary nitrogen) and functional parameters (for example hand-grip strength and the Short Physical Performance Test), in addition to body composition. Furthermore, our review is the first to investigate the key variables that influence protein status in a PKU cohort. A scoping review is pertinent to our research questions as it enables the breadth of evidence to be mapped and synthesised qualitatively, and to identify gaps in current evidence where future research can focus with the intention to ultimately improve patient care.

Objective

The primary objective of this scoping review is to examine existing evidence regarding the protein status of people with PKU and identify key nutritional and lifestyle variables that influence protein status in people with PKU.

METHODS AND ANALYSIS

Protocol design

The protocol of the proposed scoping review was informed by Arksey and O’Malley’s framework involving the following five stages: (I) identifying the research question, (II) identifying relevant studies, (III) study selection, (IV) charting the data, (V) collating, summarising and reporting the results[32], with

For peer review only - http://bmjopen.bmj.com/site/about/guidelines.xhtml

consideration for Levac et al's methodological enhancements[33], guidance by Pawliuk et al[34], and the Joanna Briggs Institute (JBI) methodology for scoping reviews[35].

Stage 1: Identifying the research question

Through consultation with the research team, the key elements of the JBI PPC mnemonic (Population, Concept and Context) were established for this review (see Eligibility criteria)[35]. These elements are reflected in the main research question and informed the inclusion and exclusion criteria.

The main research question for this scoping review is: What is the existing evidence of the protein status of people with PKU across the lifespan? For this review, the assessment of protein status encompasses biochemical, functional and anthropometric measurements, as outlined in Table 1.

In addition to this main question, the review will focus on the following sub-questions:

1. How is protein status assessed in people with PKU?
2. Which variables, known to influence protein status in the general population, have been investigated when assessing protein status in people with PKU?
3. Which variables, known to influence protein status in the general population, modulate protein status in people with PKU?

Stage 2: Identifying relevant studies

Information sources

The following databases will be searched to identify relevant literature: MEDLINE (Ovid), Embase (Ovid), CENTRAL, Web of Science and Scopus. Conference proceedings and abstracts sited in Embase (Ovid) will be examined. Unpublished studies and grey literature will be sought by contacting experts in Inherited Metabolic Diseases specialist groups and sourced from reports available from the Society for the Study of Inborn Errors of Metabolism (Journal of Inherited Metabolic Disease) and International Congress of Inborn Errors of Metabolism (Journal of Inborn Errors of Metabolism & Screening) international meetings from 2015-2020. Hand searching of the reference lists of articles selected for full text review will be undertaken to identify any additional literature. Articles published in English only will be included. No date restriction will be applied to the searches.

Search strategy

A preliminary search was undertaken by the research team to identify key words in titles and abstracts from relevant literature, and along with the index terms, were used to develop a full list of 93 search terms to encompass 'protein status'. From this, a list of 16 key terms was agreed on by the research team to be included in the full search strategy. The search strategy was developed with guidance from a Librarian and piloted in MEDLINE (Ovid) by the research team, see online supplemental material.

For peer review only - http://bmjopen.bmj.com/site/about/guidelines.xhtml

Eligibility criteria

As outlined in the JBI methodology for scoping reviews[35], establishing clear inclusion criteria is important in defining the scope of the review and to guide the research team when identifying relevant studies to include. The JBI PCC strategy was used to develop the following inclusion and exclusion criteria:

Population

This scoping review will consider studies that include participants diagnosed with PKU. Studies will be excluded if the study population incorporates the following: (1) females with PKU who are pregnant, as dietary management and phenylalanine targets can differ during pre-conception, pregnancy and post-partum; (2) people with raised phenylalanine levels not related to phenylalanine hydroxylase deficiency, and (3) people with other co-morbidities that could influence protein intake, protein digestion and amino acid absorption, such as pancreatic insufficiency, coeliac disease, irritable bowel syndrome, inflammatory bowel disease, diabetes, cancer and/or having had a gastric bypass. All age categories will be considered and defined as 'children' <18 years of age, 'adults' ≥18 years of age, and 'older adults' >60 years of age.

Concept

This scoping review will evaluate studies that report the biochemical, functional and/or anthropometric measurements of protein status in people with PKU, as outlined in Table 1. Where analysis of overall nutritional status has been reported, only measurements of protein status will be considered. Studies that report dietary protein intake without protein status outcome measurements, or those reporting only body weight, height and body mass index, will be excluded.

Table 1: Measurements of protein status considered in the review

**Anthropometric**	Body composition (fat free mass, fat mass, total body water) via dual-energy X-ray absorptiometry, bioelectrical impedance analysis, total body electrical conductivity, BodPod whole body air-displacement plethysmography, skinfolds
**Biochemical**	3-methylhistidine concentrations, albumin, pre-albumin, transthyretin, retinol-binding protein, urea production, blood urea nitrogen, urinary nitrogen, total body nitrogen, whole-body protein metabolism, and plasma amino acids concentrations, urea production and creatinine (where the author(s) have specifically used these as a measure of protein status)
**Functional**	Hand-grip strength, the Short Physical Performance Test (SPPT; including tests of balance, gait speed, and timed sit-to-stand), one repetition max (1RM, or a five-repetition max for older adults) and VO_2max testing (or VO_2peak for older adults) and other validated measures of muscle function.

For peer review only - http://bmjopen.bmj.com/site/about/guidelines.xhtml

As detailed previously, numerous variables influence protein status in the general population, including total daily protein intake, protein dose consumed on a per meal basis, the daytime distribution pattern of protein intake and in relation to exercise, and protein source; all of which warrant consideration when assessing protein status in PKU. An evaluation of whether these variables have been controlled for by the relevant studies, and the impact of these variables on protein status in people with PKU, will be included in this review. Although lifelong treatment is recommended in the management of PKU, some adolescents and adults may choose to have periods of time on an unrestricted diet. For the purpose of this review, 'on diet' is defined as restricting dietary protein and/or taking at least one protein substitute daily, and 'off diet' is defined as eating freely without restriction and not taking protein substitutes. During periods 'off diet', total dietary protein intake and the consumption of high biological protein could increase, and the proportion of total protein provided by L-AA or CGMP could decrease. This review will explore studies where participants are on a phenylalanine-restricted diet as well as those who may have had periods on and off diet, as the impact of changes in total protein intake on and off diet on protein status warrants consideration. Socioeconomic status indicators will also be extracted.

Context

This review will consider studies that report protein status in people with PKU from an international perspective. Although the existing literature base on PKU is relatively limited given that PKU is a rare condition, the inclusion of all geographical areas will ensure all available evidence can be considered. Dietary management differs between countries, and therefore consideration will be given to the protein requirements and management guidelines specific to each population included in the review.

Stage 3: Study selection

The study selection will be undertaken in accordance to the Preferred Reporting Items for Systematic Reviews and Meta-analyses extension for scoping reviews (PRISMA-ScR) flow diagram[36] (see Figure 1) and presented in the final scoping review. Following the search, all identified records will be uploaded into Zotero 5.0 (George Mason University, USA) and duplicates removed automatically and manually. The review process will consist of two stages. First, titles and abstracts will be screened by two independent reviewers to determine potential eligibility. Second, the full text of records deemed relevant will be retrieved and assessed in detail against the inclusion criteria by the same two independent reviewers. As recommended by Pawliuk et al[34], the study selection process will be tested using 5% of the articles identified, prior to initiating the formal scoping review study selection, to ensure the reviewers understand the inclusion criteria. The two reviewers will meet to resolve any disagreements that arise at each stage of the study selection process, and where consensus cannot be reached, through discussions with a secondary review panel.

Stage 4: Charting the data

Data will be charted by two independent reviewers using an adapted version of the JBI extraction instrument[35]. The key data to be extracted from the selected articles are outlined in Box 1 and will

For peer review only - http://bmjopen.bmj.com/site/about/guidelines.xhtml

include details about the population, concept, context and information relevant to the review questions. The draft data charting tool will be piloted for purpose, and modified as required. Once the first five articles have been charted by the two reviewers independently, the reviewers will meet to ensure there is consistency in information extracted.

Stage 5: Collating, summarising and reporting the results

The extracted data will be tabulated in a manner that aligns with the research objectives and questions of this scoping review. This table will provide a descriptive numerical summary of the study characteristics including the total number of studies involved, year of publication, study design, study population characteristics, and primary outcomes. A further descriptive numerical summary will outline the measurements of protein status used and the findings from the studies (including any descriptive statistics and effect size where available). Due to existing concerns regarding whether current guidelines on protein requirements are adequate to offset age-related changes in protein metabolism across the adult lifespan[7], in addition to reporting the results on protein status across the lifespan, a summary of evidence from research specific to adults and older adults will be included.

To address the sub-questions on the variables considered and the impact of variables on protein status, the variables controlled for in each study will be tabulated alongside the study's results of protein status to map findings of any correlations of these variables with protein status in people with PKU. This exercise will identify gaps in current research where variables known to influence protein status in the general population need further consideration when determining protein recommendations for PKU dietary management guidelines. A narrative summary will accompany the tabulated results and will describe how the results relate to the review's objective and questions. The PRISMA-ScR will guide the reporting of the results[36].

Patient and public involvement

There is no patient and public involvement in the design or conduct of the review. The review findings will be shared with a patient and public involvement group, established to advise on research in PKU, to inform discussions for the direction of future research.

ETHICS AND DISSEMINATION

As this study is based on the review of publicly available information, the review does not require ethical approval. This review is the first step in a larger research project to contribute to the development of robust evidence-based protein recommendation guidelines for individuals with PKU. Findings from this review will be disseminated through peer-reviewed publication, and shared at conferences and national meetings on Inherited Metabolic Disease. As outlined in 'Patient and public involvement', the review will also be shared with a patient group established to advise on research in PKU to inform plans for further research.

For peer review only - http://bmjopen.bmj.com/site/about/guidelines.xhtml

Box I: Key data to be extracted

1. Author(s)
2. Year of publication
3. Title and journal
4. Country of origin
5. Aims/purpose
6. Population characteristics (sample size, age, sex, ethnicity, time of diagnosis, socioeconomic status)
7. Methodology / methods
8. Intervention type, comparator and details of these (if applicable). Duration of the intervention (if applicable)
9. Primary outcome
10. Protein status outcomes and how these were measured
    a. Anthropometric
    b. Biochemical
    c. Functional
11. Variables controlled for in the study
12. Key findings that relate to the review questions
13. Study limitations

Insert Figure 1: Preferred Reporting Items for Systematic Reviews and Meta-Analysis flow diagram for scoping review process. Retrieved from Tricco et al.

For peer review only

For peer review only - http://bmjopen.bmj.com/site/about/guidelines.xhtml

Author Contributions

All authors were involved in conceptualisation, development and design of protocol methodology and critical revision of the draft and final manuscript. SF prepared the original draft. MO was lead supervisor for the development of the protocol. All authors approved the final manuscript.

Acknowledgments

The Library Services at King's College London for their guidance in the development of the search strategy.

Conflict of interests

Sarah Firman has received funding to attend conferences and study days from Nutricia, Vitaflo International and Dr. Schär UK Ltd, and consulting fees from Vitaflo International and Meta Healthcare Ltd. Kevin Whelan is in receipt of research funding from Danone and has acted as a consultant for Danone.

Funding

Sarah Firman is funded by Health Education England/National Institute for Health Research (NIHR300395). This review presents independent research funded by the HEE/NIHR. The views expressed are those of the author(s) and not necessarily those of HEE, the NHS, the NIHR or the Department of Health and Social Care.

For peer review only

For peer review only - http://bmjopen.bmj.com/site/about/guidelines.xhtml

REFERENCES

1. Singh RH, Cunningham AC, Mofidi S, et al. Updated, web-based nutrition management guideline for PKU: An evidence and consensus based approach. *Mol Genet Metab*2016;118(2):72–83.

2. van Wegberg AMJ, MacDonald A, Ahring K, et al. The complete European guidelines on phenylketonuria: Diagnosis and treatment. *Orphanet J Rare Dis*2017 Oct;12(1).

3. Inwood A, Lewis K, Balasubramaniam S, et al. Australasian consensus guidelines for the management of phenylketonuria (PKU) throughout the lifespan [Internet]. 2017. p. 1–36. Available from: https://www.mdda.org.au/resources/handbooks-guidelines/

4. Institute of Medicine. Dietary Reference Intakes for Energy, Carbohydrate, Fiber, Fat, Fatty Acids, Cholesterol, Protein, and Amino Acids. Dietary Reference Intakes for Energy, Carbohydrate, Fiber, Fat, Fatty Acids, Cholesterol, Protein, and Amino Acids (Macronutrients). Washington, D.C: National Academies Press; 2005.

5. National Health and Medical Research Council. Nutrient Reference Values for Australia and New Zealand Including Recommended Dietary Intakes. Canberra: NHMRC; 2006.

6. Food and Agriculture Organization of the United Nations, World Health Organization & United Nations University. Protein and amino acid requirements in human nutrition: report of a joint FAO/WHO/UNU expert consultation. World Health Organization technical report series. Geneva, Switzerland: World Health Organization; 2007.

7. Firman S, Witard OC, O'Keeffe M, Ramachandran R. Dietary protein and protein substitute requirements in adults with phenylketonuria: A review of the clinical guidelines. *Clin Nutr*2020 Nov 11. http://www.sciencedirect.com/science/article/pii/S0261561420306075 (cited 2020 Nov 17)

8. Rand WM, Pellett PL, Young VR. Meta-analysis of nitrogen balance studies for estimating protein requirements in healthy adults. *Am J Clin Nutr*2003;77(1):109–27.

9. Rodriguez N, Miller S. Effective translation of current dietary guidance: Understanding and communicating the concepts of minimal and optimal levels of dietary protein. *Am J Clin Nutr*2015;101(6):1353S-1358S.

10. Rodriguez NR. Introduction to Protein Summit 2.0: Continued exploration of the impact of high-quality protein on optimal health. *Am J Clin Nutr*2015;101(6):1317S-1319S.

11. Gropper SS, Acosta PB. Effect of simultaneous ingestion of L-amino acids and whole protein on plasma amino acid and urea nitrogen concentrations in humans. *J Parenter Enter Nutr*1991;15(1):48–53.

For peer review only - http://bmjopen.bmj.com/site/about/guidelines.xhtml

12. Metges CC, El-Khoury AE, Selvaraj AB, et al. Kinetics of L-[1-13C]leucine when ingested with free amino acids, unlabeled or intrinsically labeled casein. *Am J Physiol Endocrinol Metab*2000;278(6 41-6):1000–9.

13. Dangin M, Boirie Y, Guillet C, et al. Influence of the protein digestion rate on protein turnover in young and elderly subjects. *J Nutr*2002;132(10):3228–33.

14. Dangin M, Boirie Y, Garcia-Rodenas C, et al. The digestion rate of protein is an independent regulating factor of postprandial protein retention. *Am J Physiol Endocrinol Metab*2001;280(2 43-2):340–8.

15. Boirie Y, Dangin M, Gachon P, et al. Slow and fast dietary proteins differently modulate postprandial protein accretion. *Proc Natl Acad Sci U S A*1997;94(26):14930–5.

16. Witard O, Wardle S, Macnaughton L, et al. Protein Considerations for Optimising Skeletal Muscle Mass in Healthy Young and Older Adults. *Nutrients*2016 Mar 23;8(4):181.

17. Paddon-Jones D, Campbell WW, Jacques PF, et al. Protein and healthy aging. *Am J Clin Nutr*2015;101(6):1339S-1345S.

18. Moore DR, Areta J, Coffey VG, et al. Daytime pattern of post-exercise protein intake affects whole-body protein turnover in resistance-trained males. *Nutr Metab*2012;9(1):91.

19. Liao CD, Tsauo JY, Wu YT, et al. Effects of protein supplementation combined with resistance exercise on body composition and physical function in older adults: a systematic review and meta-analysis. *Am J Clin Nutr*2017 Oct;106(4):1078–91.

20. Witard OC, Jackman SR, Breen L, et al. Myofibrillar muscle protein synthesis rates subsequent to a meal in response to increasing doses of whey protein at rest and after resistance exercise. *Am J Clin Nutr*2014 Jan;99(1):86–95.

21. Shad BJ, Thompson JL, Holwerda AM, et al. One Week of Step Reduction Lowers Myofibrillar Protein Synthesis Rates in Young Men. *Med Sci Sports Exerc*2019;51(10):2125–34.

22. Smith GI, Reeds DN, Hall AM, et al. Sexually dimorphic effect of aging on skeletal muscle protein synthesis. *Biol Sex Differ*2012 May 23;3(1):11.

23. Moore DR, Churchward-Venne TA, Witard O, et al. Protein ingestion to stimulate myofibrillar protein synthesis requires greater relative protein intakes in healthy older versus younger men. *J Gerontol A Biol Sci Med Sci*2015;70(1):57–62.

24. Cuthbertson D, Smith K, Babraj J, et al. Anabolic signaling deficits underlie amino acid resistance of wasting, aging muscle. *FASEB J*2005;19(3):1–22.

For peer review only - http://bmjopen.bmj.com/site/about/guidelines.xhtml

25. Burd NA, Tang JE, Moore DR, et al. Exercise training and protein metabolism: influences of contraction, protein intake, and sex-based differences. *J Appl Physiol Bethesda Md*1985. 2009 May;106(5):1692–701.

26. Allen JR, Baur LA, Waters DL, et al. Body protein in prepubertal children with phenylketonuria. *Eur J Clin Nutr*1996 Mar;50(3):178–86.

27. Doulgeraki A, Skarpalezou A, Theodosiadou A, et al. Body composition profile of young patients with phenylketonuria and mild hyperphenylalaninemia. *J Endocrinol Metab*2014 Jul;12(3):e16061.

28. Evans M, Truby H, Boneh A. The relationship between dietary intake, growth and body composition in Phenylketonuria. *Mol Genet Metab*2017;122(1–2):36–42.

29. Mazzola PN, Nalin T, Castro K, et al. Analysis of body composition and nutritional status in Brazilian phenylketonuria patients. *Mol Genet Metab Rep*2016 Mar;1:16–20.

30. Acosta PB, Yannicelli S, Marriage B, et al. Protein status of infants with phenylketonuria undergoing nutrition management. *J Am Coll Nutr*1999 Apr;18(2):102–7.

31. PROSPERO: International prospective register of systematic reviews. Search term: Phenylketonuria. Available from: https://www.crd.york.ac.uk/prospero/#searchadvanced (accessed 01 Feb 2021)

32. Arksey H, O'Malley L. Scoping studies: towards a methodological framework. *Int J Soc Res Methodol*2005 Feb;8(1):19–32.

33. Levac D, Colquhoun H, O'Brien KK. Scoping studies: advancing the methodology. *Implement Sci*2010 Dec;5(1):69.

34. Pawliuk C, Brown HL, Widger K, et al. Optimising the process for conducting scoping reviews. *BMJ Evid-Based Med*2020 Oct 21;bmjebm-2020-111452. doi: 10.1136/bmjebm-2020-111452.

35. Peters MDJ, Godfrey C, McInerney P, Munn Z, Tricco AC, Khalil, H. Chapter 11: Scoping Reviews (2020 version). In: Aromataris E, Munn Z (Editors). JBI Manual for Evidence Synthesis, JBI, 2020. Available from https://synthesismanual.jbi.global. https://doi.org/10.46658/JBIMES-20-12

36. Tricco AC, Lillie E, Zarin W, et al. PRISMA Extension for Scoping Reviews (PRISMA-ScR): Checklist and Explanation. *Ann Intern Med*2018 Sep 4;169(7):467–73.

For peer review only - http://bmjopen.bmj.com/site/about/guidelines.xhtml

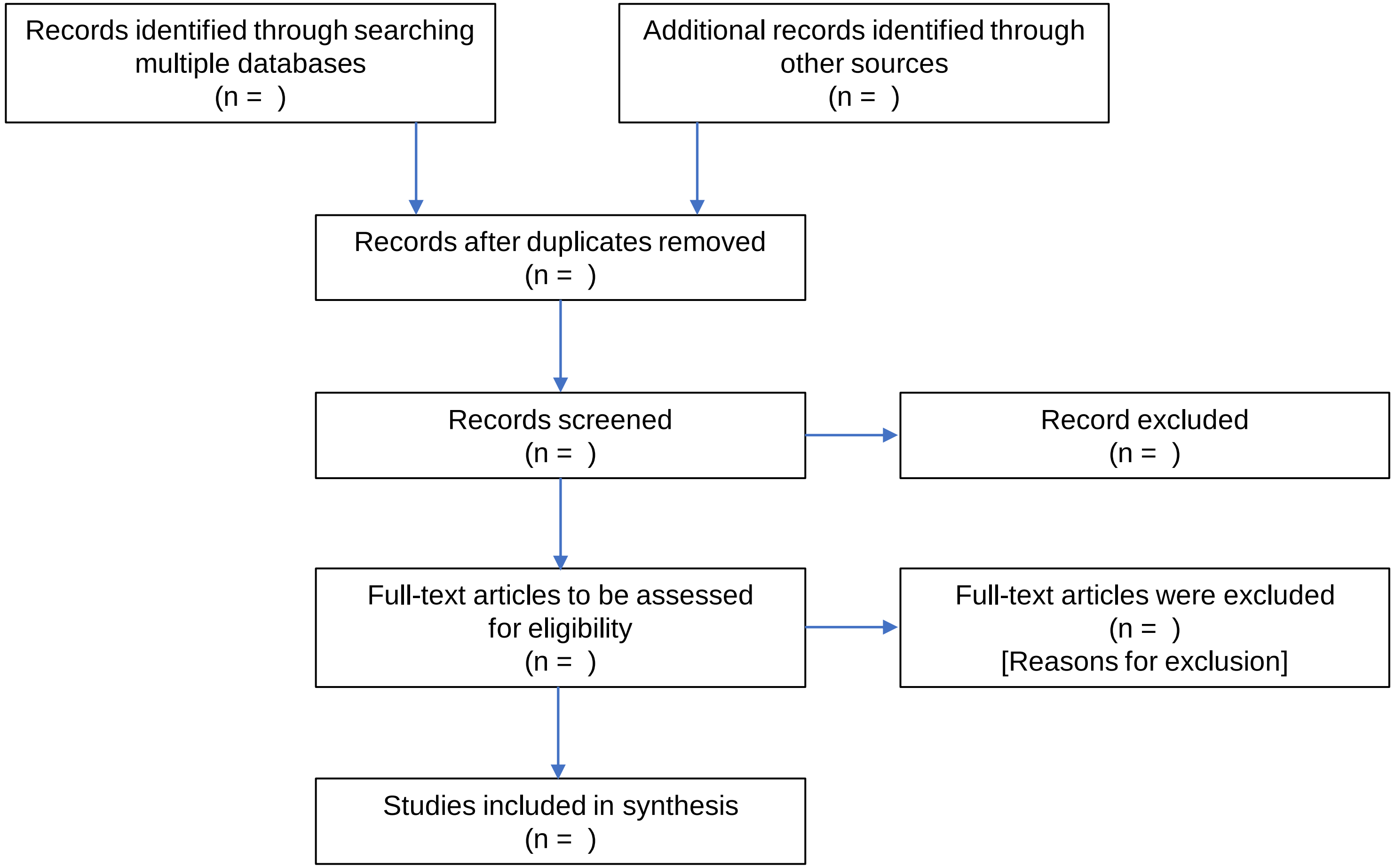

Figure 1: Preferred Reporting Items for Systematic Reviews and Meta-Analysis flow diagram for scoping review process. Retrieved from Tricco et al.

Supplemental material: Search strategy

Database: MEDLINE (Ovid) < 1946 to January 15, 2021>

Search	Query	Records retrieved
#1	exp Phenylketonurias/ OR Phenylketonurias.mp. OR PKU.mp. OR Hyperphenylalanin?emia.mp. OR exp Phenylalanine Hydroxylase/ OR "Phenylalanine Hydroxylase".mp. OR "Phenylalanine Hydroxylase deficiency".mp.	8868
#2	exp Nutritional Status/ OR "nutritional status".mp. OR "protein status".mp. OR exp Muscle Proteins/ OR "muscle proteins".mp. OR "protein metabolism".mp. OR "muscle protein metabolism".mp. OR exp Body Composition/ OR "body composition".mp. OR exp Muscle Strength/ OR "muscle strength".mp. OR "muscle function".mp. OR exp Prealbumin/ OR prealbumin.mp. OR Transthyretin.mp. OR exp Albumins/ OR albumin.mp. OR "3-methylhistidine concentrations".mp. OR exp Retinol-Binding Proteins/ OR "retinol-binding protein".mp. OR "urea production".mp. OR exp Nitrogen/ OR nitrogen.mp. OR exp Creatinine/ OR creatinine.mp.	959815
#3	#1 AND #2	**272**
Limit	English only	**246**

For peer review only

For peer review only - http://bmjopen.bmj.com/site/about/guidelines.xhtml

BMJ Open

Protein status of people with phenylketonuria: A scoping review protocol

Journal:	*BMJ Open*
Manuscript ID	bmjopen-2021-049883.R1
Article Type:	Protocol
Date Submitted by the Author:	01-Jun-2021
Complete List of Authors:	Firman, Sarah; King's College London, Department of Nutritional Sciences; Guy's and St Thomas' NHS Foundation Trust, Adult Inherited Metabolic Diseases<br>Ramachandran, Radha; Guy's and St Thomas' NHS Foundation Trust, Adult Inherited Metabolic Diseases<br>Whelan, Kevin; King's College London, Department of Nutritional Sciences<br>Witard, Oliver ; King's College London, Department of Nutritional Sciences; King's College London, Centre for Human and Applied Physiological Sciences<br>O'Keeffe, Majella; King's College London, Department of Nutritional Sciences; University College Cork, School of Food and Nutritional Sciences
**Primary Subject Heading**:	Nutrition and metabolism
Secondary Subject Heading:	Research methods
Keywords:	Protocols & guidelines < HEALTH SERVICES ADMINISTRATION & MANAGEMENT, NUTRITION & DIETETICS, GENETICS

SCHOLARONE™
Manuscripts

I, the Submitting Author has the right to grant and does grant on behalf of all authors of the Work (as defined in the below author licence), an exclusive licence and/or a non-exclusive licence for contributions from authors who are: i) UK Crown employees; ii) where BMJ has agreed a CC-BY licence shall apply, and/or iii) in accordance with the terms applicable for US Federal Government officers or employees acting as part of their official duties; on a worldwide, perpetual, irrevocable, royalty-free basis to BMJ Publishing Group Ltd ("BMJ") its licensees and where the relevant Journal is co-owned by BMJ to the co-owners of the Journal, to publish the Work in this journal and any other BMJ products and to exploit all rights, as set out in our licence.

The Submitting Author accepts and understands that any supply made under these terms is made by BMJ to the Submitting Author unless you are acting as an employee on behalf of your employer or a postgraduate student of an affiliated institution which is paying any applicable article publishing charge ("APC") for Open Access articles. Where the Submitting Author wishes to make the Work available on an Open Access basis (and intends to pay the relevant APC), the terms of reuse of such Open Access shall be governed by a Creative Commons licence – details of these licences and which Creative Commons licence will apply to this Work are set out in our licence referred to above.

Other than as permitted in any relevant BMJ Author's Self Archiving Policies, I confirm this Work has not been accepted for publication elsewhere, is not being considered for publication elsewhere and does not duplicate material already published. I confirm all authors consent to publication of this Work and authorise the granting of this licence.

For peer review only

Protein status of people with phenylketonuria: A scoping review protocol

Sarah Firman[1,2,3], Radha Ramachandran[3], Kevin Whelan[1], Oliver C. Witard[1,4], Majella O'Keeffe[1,5]

1. Department of Nutritional Sciences, King's College London, London, United Kingdom
2. Department of Nutrition and Dietetics, Guy's and St Thomas' NHS Foundation Trust, London, United Kingdom
3. Adult Inherited Metabolic Diseases, Guy's and St Thomas' NHS Foundation Trust, London, United Kingdom
4. Centre for Human and Applied Physiological Sciences, King's College London, London, United Kingdom
5. School of Food and Nutritional Sciences, University College Cork, College Road, Cork, Ireland

Corresponding author:

Dr Majella O'Keeffe
School of Food and Nutritional Sciences
Biosciences Institute, Room 4.08
University College Cork
Cork
Ireland
Email: majella.okeeffe@ucc.ie

Word count: 2614 words

For peer review only - http://bmjopen.bmj.com/site/about/guidelines.xhtml

ABSTRACT

Introduction: Phenylketonuria (PKU) is a disorder of protein metabolism resulting in an accumulation of phenylalanine in the body. Dietary management consists of altering the sources of ingested protein to limit phenylalanine intake. Current dietary protein guidelines for PKU are based on limited scientific evidence, thus it remains unclear whether current practice leads to optimal protein status in people with PKU. To date, no attempt has been made to systematically evaluate the protein status of people with PKU, using a combination of validated anthropometric, biochemical and functional measurement tools. Furthermore, factors known to influence protein status in the general population warrant consideration when determining protein status in individuals with PKU, alongside factors unique to PKU such as the type of protein substitute consumed. Understanding the impact of these variables on protein status is crucial to developing a personalised approach to protein recommendations for optimising health and functional outcomes in people with PKU. Therefore, the aim of this scoping review is to examine existing evidence regarding the protein status of people with PKU, and to investigate the nutritional and lifestyle variables that influence protein status.

Methods and analysis: This review will be guided by Arksey and O'Malley's framework, along with guidance from Levac et al, Pawliuk et al, and the Joanna Briggs Institute. The following databases will be searched: MEDLINE(Ovid), Embase, CENTRAL, Web of Science and Scopus, alongside grey literature. Identified literature will be assessed by two independent reviewers for inclusion. Descriptive numerical analysis will be performed and a narrative summary will accompany the tabulated results describing how study findings relate to the review questions.

Ethics and dissemination: This review protocol does not require ethical approval. Findings will be disseminated through peer-reviewed publication, presented at relevant conferences, and shared with a patient research advisory group to inform discussions on future research.

Keywords: body composition; muscle function; phenylketonuria; protein blood levels; protein status

For peer review only

For peer review only - http://bmjopen.bmj.com/site/about/guidelines.xhtml

ARTICLE SUMMARY

Strengths and limitations of this study

- This review is the first to collate data from various anthropometric, biochemical and functional measurements of protein status in people with PKU.
- With limited research in the area of PKU, the broad research question will allow for the mapping of available evidence, and to identify the gaps in existing research to inform the direction of further research into improving the care provided for people with PKU.
- The search strategy includes identification of published and unpublished literature, including sourcing grey literature by contacting specialist groups and hand searching conference and meeting reports.
- The protocol includes a clearly defined inclusion criteria aligned with Joanna Briggs Institute PCC strategy to ensure transparency of information sourced for evaluation in the review.
- Aligned with the accepted framework for scoping reviews, this study does not include formal assessment of the quality of evidence.

List of Abbreviations:

CGMP	Casein glycomacropeptide
JBI	Joanna Briggs Institute
L-AA	L-amino acids
OMIM	Online Mendelian Inheritance in Man
PCC	Population, Concept, and Context
PKU	Phenylketonuria
PRISMA-ScR	Preferred Reporting Items for Systematic Reviews and Meta-analyses extension for scoping reviews
SPPT	Short Physical Performance Test
1RM	One repetition maximum

For peer review only

For peer review only - http://bmjopen.bmj.com/site/about/guidelines.xhtml

INTRODUCTION

Phenylketonuria (PKU; OMIM 261600) is an inborn error of protein metabolism that leads to an accumulation of phenylalanine in the blood and brain. Early diagnosis and treatment are essential in preventing profound and irreversible intellectual disability, in addition to reducing other neurological consequences associated with high phenylalanine throughout life. Lifelong treatment is recommended for individuals with blood phenylalanine levels above specific phenylalanine targets, which can vary depending on the management guidelines adopted[1–3]. Treatment should be started according to country-specific guidelines or, where guidelines are not available, to European or United States consensus guidelines[1–3]. Currently, dietary management provides the mainstay of treatment and consists of altering the sources of ingested protein to limit phenylalanine intake, and supplementation with phenylalanine-free or low-phenylalanine protein sources to meet protein requirements[4]. In practice, this dietary regimen involves restricting protein intake from food and instead providing the majority (up to 80%) of protein intake from phenylalanine-free or low-phenylalanine protein substitutes, consisting of either L-amino acids (L-AA) or casein glycomacropeptide (CGMP)[5,6], and more recently, protein substitutes utilising prolonged-release amino acid technology[7–9].

A key limitation of existing guidelines on dietary protein requirements for people with PKU[1–3] is that the data has been extrapolated from studies that estimated protein requirements in healthy populations[4]. These studies utilised nitrogen balance methodology to evaluate the minimum protein (i.e. nitrogen) intake required to balance nitrogen losses at the whole-body level of physiology[10]. In contrast to the definition of a protein requirement, a protein recommendation serves to optimise metabolic function and improve health and functional outcomes in a given population group[11,12]. Experimental studies conducted in healthy young (18-35 year old) adults without PKU provide useful insight into the impact of L-AA supplementation on plasma amino acid kinetics[13–17]. The rapid rise in plasma amino acid concentrations observed with ingestion of L-AA has been shown to result in greater amino acid oxidation rates and lower protein retention at the whole-body level in comparison with whole protein. To account for the reduced uptake and utilisation of amino acids from protein substitutes, current advice is to apply a correction factor of 20-40% in excess of the protein requirement guidelines of the general population[1–3]. However, the evidence underpinning protein requirement guidelines for dietary management of adults with PKU is limited, and in their current form may not adequately offset age-related changes in protein metabolism across the adult lifespan in people with PKU[4].

In addition to total daily protein intake, multiple factors are known to influence protein status in the non-PKU population. These factors include, but are not limited to, the protein dose ingested per meal/serving, protein source, the timing of protein intake in relation to physical activity and/or other nutrients, the daytime distribution pattern of protein intake, and also the co-ingestion of other nutrients alongside protein[18–24]. Furthermore, age, sex and physical activity status are all known to modify the metabolic fate of ingested protein[23–27]. These variables are also relevant when considering research undertaken to determine protein status in individuals with PKU. In addition, there are factors unique to the dietary regimen of people with PKU that warrant consideration such as type of protein substitute (L-

For peer review only - http://bmjopen.bmj.com/site/about/guidelines.xhtml

AA versus CGMP versus protein substitutes utilising prolonged release technologies), dose and timing of protein substitute ingestion, the proportion of total protein intake from dietary protein versus protein substitutes, adherence to protein substitute prescription, and the period of time spent on and off a phenylalanine restricted diet.

It remains unclear whether existing protein requirement guidelines for people with PKU translate to optimal protein status across their lifespan. Developing an understanding of the variables that modulate protein status, both positively and negatively, will be crucial to informing future research on personalising protein recommendations for individuals of all ages with PKU, with the intention of improving the care provided for people with PKU. The term ‘protein status’ encompasses a vast range of measurements and physiological outcomes, and for the purpose of this scoping review the term will include those parameters detailed in Table 1. Studies investigating body composition and biochemical measurements of protein nutritional status have been conducted in children and adults with PKU[28–34], yet no contemporary synthesis has been undertaken. Mapping the available evidence is important to identify the direction(s) for future research into establishing evidence-based protein recommendations for dietary management of PKU. In this regard, a scoping review provides the ideal approach to explore the breadth of literature on the protein status of people with PKU in order to map and synthesise the evidence and identify the key variables that influence protein status in this population.

To date, no attempt has been made to systematically evaluate the protein status of people with PKU, using a combination of validated anthropometric, biochemical and functional measurement tools. Although three systematic reviews of protein status in people with PKU are currently registered on PROSPERO, two are exclusively focused on bone health (which is not the focus of the proposed review), with the other systematic review and meta-analysis investigating measurements of body composition only, in patients with PKU[35]. Our scoping review is unique in taking a holistic approach by encompassing a range of validated measurements of protein status, including biochemical (for example 3-methylhistidine concentrations, pre-albumin, and urinary nitrogen) and functional parameters (for example hand-grip strength and the Short Physical Performance Test), in addition to body composition. Furthermore, our review is the first to investigate the key variables that influence protein status in a PKU cohort across the lifespan. A scoping review is pertinent to our research questions as it enables the breadth of evidence to be mapped and synthesised qualitatively, and to identify gaps in current evidence where future research can focus with the intention to ultimately improve patient care.

Objective

The primary objective of this scoping review is to examine existing evidence regarding the protein status of people with PKU and identify key nutritional and lifestyle variables that influence protein status in people with PKU.

For peer review only - http://bmjopen.bmj.com/site/about/guidelines.xhtml

METHODS AND ANALYSIS

Protocol design

The protocol of the proposed scoping review was informed by Arksey and O'Malley's framework involving the following five stages: (I) identifying the research question, (II) identifying relevant studies, (III) study selection, (IV) charting the data, (V) collating, summarising and reporting the results[36], with consideration for Levac et al's methodological enhancements[37], guidance by Pawliuk et al[38], and the Joanna Briggs Institute (JBI) methodology for scoping reviews[39].

Stage 1: Identifying the research question

Through consultation with the research team, the key elements of the JBI PPC mnemonic (Population, Concept and Context) were established for this review (see Eligibility criteria)[39]. These elements are reflected in the main research question and informed the inclusion and exclusion criteria.

The main research question for this scoping review is: What is the existing evidence of the protein status of people with PKU across the lifespan? For this review, the assessment of protein status encompasses biochemical, functional and anthropometric measurements, as outlined in Table 1.

In addition to this main question, the review will focus on the following sub-questions:

1. How is protein status assessed in people with PKU?
2. Which variables, known to influence protein status in the general population, have been investigated when assessing protein status in people with PKU?
3. Which variables, known to influence protein status in the general population, modulate protein status in people with PKU?

Stage 2: Identifying relevant studies

Information sources

The following databases will be searched to identify relevant literature: MEDLINE (Ovid), Embase (Ovid), CENTRAL, Web of Science and Scopus. Conference proceedings and abstracts sited in Embase (Ovid) will be examined. Unpublished studies and grey literature will be sought by contacting experts in Inherited Metabolic Diseases specialist groups and sourced from reports available from the Society for the Study of Inborn Errors of Metabolism (Journal of Inherited Metabolic Disease) and International Congress of Inborn Errors of Metabolism (Journal of Inborn Errors of Metabolism & Screening) international meetings from 2010-2020. Hand searching of the reference lists of articles selected for full text review will be undertaken to identify any additional literature. Articles published in English only will be included. No date restriction will be applied to the searches. The search to identify relevant literature will be conducted between June and July 2021.

For peer review only - http://bmjopen.bmj.com/site/about/guidelines.xhtml

Search strategy

A preliminary search was undertaken by the research team to identify key words in titles and abstracts from relevant literature, and along with the index terms, were used to develop a full list of 96 search terms to encompass 'protein status'. From this, a list of 19 key terms were agreed on by the research team to be included in the full search strategy. The search strategy was developed with guidance from a Librarian and piloted in MEDLINE (Ovid) by the research team, see online supplemental material.

Eligibility criteria

As outlined in the JBI methodology for scoping reviews[39], establishing clear inclusion criteria is important in defining the scope of the review and to guide the research team when identifying relevant studies to include. The JBI PCC strategy was used to develop the following inclusion and exclusion criteria:

Population

This scoping review will consider studies that include participants diagnosed with PKU. Studies will be excluded if the study population incorporates the following: (1) females with PKU who are pregnant, as dietary management and phenylalanine targets can differ during pre-conception, pregnancy and post-partum; and (2) people with other co-morbidities that could influence protein intake, protein digestion and amino acid absorption, such as pancreatic insufficiency, coeliac disease, irritable bowel syndrome, inflammatory bowel disease, diabetes, cancer and/or having had a gastric bypass. All age categories will be considered and defined as 'children' <18 years of age, 'adults' ≥ 18 years of age, and 'older adults' >60 years of age.

Concept

This scoping review will evaluate studies that report the biochemical, functional and/or anthropometric measurements of protein status in people with PKU, as outlined in Table 1. Where analysis of overall nutritional status has been reported, only measurements of protein status will be considered. Studies that report dietary protein intake without protein status outcome measurements, or those reporting only body weight, height and body mass index, will be excluded.

Table 1: Measurements of protein status considered in the review

**Anthropometric**	Body composition (fat free mass, fat mass, total body water) via dual-energy X-ray absorptiometry, bioelectrical impedance analysis, total body electrical conductivity, BodPod whole body air-displacement plethysmography, skinfolds
**Biochemical**	3-methylhistidine concentrations, albumin, pre-albumin, transthyretin, retinol-binding protein, urea production, blood urea nitrogen, urinary nitrogen, total body nitrogen, whole-body protein metabolism, and plasma

For peer review only - http://bmjopen.bmj.com/site/about/guidelines.xhtml

	amino acids concentrations, urea production and creatinine (where the author(s) have specifically used these as a measure of protein status)
**Functional**	Hand-grip strength, the Short Physical Performance Test (SPPT; including tests of balance, gait speed, and timed sit-to-stand), one repetition max (1RM, or a five-repetition max for older adults) and VO_2max testing (or VO_2peak for older adults) and other validated measures of muscle function (i.e. isokinetic quadriceps strength using dynamometry and vertical jump performance using force platform technology).

As detailed previously, numerous variables influence protein status in the general population, including total daily protein intake, protein dose consumed on a per meal basis, the daytime distribution pattern of protein intake and in relation to exercise, and protein source, in addition to age, sex and physical activity status; all of which warrant consideration when assessing protein status in PKU. Furthermore, factors relevant to PKU dietary management such as the proportion of total protein intake from dietary protein versus protein substitutes, type of protein substitute (L-AA versus CGMP versus prolonged release technologies), adherence to protein substitute prescription and metabolic control warrant consideration. An evaluation of whether these variables have been controlled for by the relevant studies, and the impact of these variables on protein status in people with PKU, will be included in this review.

Although lifelong treatment is recommended in the management of PKU, some adolescents and adults may choose to have periods of time on an unrestricted diet. If available, information on participants' history with the PKU diet will be obtained, as the impact of changes in total protein intake on and off diet on protein status warrants consideration. Socioeconomic status indicators will also be extracted.

Context

This review will consider studies that report protein status in people with PKU from an international perspective. Although the existing literature base on PKU is relatively limited given that PKU is a rare condition, the inclusion of all geographical areas will ensure all available evidence can be considered. Dietary management differs between countries, and therefore consideration will be given to the protein requirements and management guidelines specific to each population included in the review.

Stage 3: Study selection

The study selection will be undertaken in accordance to the Preferred Reporting Items for Systematic Reviews and Meta-analyses extension for scoping reviews (PRISMA-ScR) flow diagram[40] (see Figure 1) and presented in the final scoping review. Following the search, all identified records will be uploaded into Zotero 5.0 (George Mason University, USA) and duplicates removed automatically and manually. The review process will consist of two stages. First, titles and abstracts will be screened by two independent reviewers to determine potential eligibility. Second, the full text of records deemed relevant will be retrieved and assessed in detail against the inclusion criteria by the same two independent

For peer review only - http://bmjopen.bmj.com/site/about/guidelines.xhtml

reviewers. As recommended by Pawliuk et al[38], the study selection process will be tested using 5% of the articles identified, prior to initiating the formal scoping review study selection, to ensure the reviewers understand the inclusion criteria. The two reviewers will meet to resolve any disagreements that arise at each stage of the study selection process, and where consensus cannot be reached, through discussions with a secondary review panel.

Stage 4: Charting the data

Data will be charted by two independent reviewers using an adapted version of the JBI extraction instrument[39]. The key data to be extracted from the selected articles are outlined in Box 1 and will include details about the population, concept, context and information relevant to the review questions. The draft data charting tool will be piloted for purpose, and modified as required. Once the first five articles have been charted by the two reviewers independently, the reviewers will meet to ensure there is consistency in information extracted.

Stage 5: Collating, summarising and reporting the results

The extracted data will be tabulated in a manner that aligns with the research objectives and questions of this scoping review. This table will provide a descriptive numerical summary of the study characteristics including the total number of studies involved, year of publication, study design, study population characteristics, and primary outcomes. A further descriptive numerical summary will outline the measurements of protein status used and the findings from the studies (including any descriptive statistics and effect size where available). Due to existing concerns regarding whether current guidelines on protein requirements are adequate to offset age-related changes in protein metabolism across the adult lifespan[4], in addition to reporting the results on protein status across the lifespan, a summary of evidence from research specific to adults and older adults will be included.

To address the sub-questions on the variables considered and the impact of variables on protein status, the variables controlled for in each study will be tabulated alongside the study's results of protein status to map findings of any correlations of these variables with protein status in people with PKU. This exercise will identify gaps in current research where variables known to influence protein status in the general population need further consideration when determining protein recommendations for PKU dietary management guidelines. A narrative summary will accompany the tabulated results and will describe how the results relate to the review's objective and questions. The PRISMA-ScR will guide the reporting of the results[40].

Patient and public involvement

For peer review only - http://bmjopen.bmj.com/site/about/guidelines.xhtml

There is no patient and public involvement in the design or conduct of the review. The review findings will be shared with a patient and public involvement group, established to advise on research in PKU, to inform discussions for the direction of future research.

ETHICS AND DISSEMINATION

As this study is based on the review of publicly available information, the review does not require ethical approval. This review is the first step in a larger research project to contribute to the development of robust evidence-based protein recommendation guidelines for individuals with PKU. Findings from this review will be disseminated through peer-reviewed publication, and shared at conferences and national meetings on Inherited Metabolic Disease. As outlined in 'Patient and public involvement', the review will also be shared with a patient group established to advise on research in PKU to inform plans for further research.

For peer review only

For peer review only - http://bmjopen.bmj.com/site/about/guidelines.xhtml

Box I: Key data to be extracted

1. Author(s)
2. Year of publication
3. Title and journal
4. Country of origin
5. Aims/purpose
6. Population characteristics (sample size, age, sex, ethnicity, time of diagnosis, metabolic control, genotype, socioeconomic status)
7. Methodology / methods
8. Intervention type, comparator and details of these (if applicable). Duration of the intervention (if applicable)
9. Primary outcome
10. Protein status outcomes and how these were measured
    a. Anthropometric
    b. Biochemical
    c. Functional
11. Variables controlled for in the study
12. Key findings that relate to the review questions
13. Study limitations

Insert Figure 1: Preferred Reporting Items for Systematic Reviews and Meta-Analysis flow diagram for scoping review process. Retrieved from Tricco et al.

For peer review only - http://bmjopen.bmj.com/site/about/guidelines.xhtml

Author Contributions

SF, RR, KW, OW and MO were involved in conceptualisation, development of the research questions and design of protocol methodology including the search strategy. SF prepared the original draft. MO was lead supervisor for the development of the protocol. RR, KW, OW and MO provided critical revision of the draft. SF, RR, KW, OW and MO revised and approved the final manuscript.

Acknowledgments

The Library Services at King's College London for their guidance in the development of the search strategy.

Conflict of interests

Sarah Firman has received funding to attend conferences and study days from Nutricia, Vitaflo International and Dr. Schär UK Ltd, and consulting fees from Vitaflo International and Meta Healthcare Ltd. Kevin Whelan is in receipt of research funding from Danone and has acted as a consultant for Danone.

Funding

Sarah Firman is funded by Health Education England/National Institute for Health Research (NIHR300395). This review presents independent research funded by the HEE/NIHR. The views expressed are those of the author(s) and not necessarily those of HEE, the NHS, the NIHR or the Department of Health and Social Care.

For peer review only

REFERENCES

1. Singh RH, Cunningham AC, Mofidi S, et al. Updated, web-based nutrition management guideline for PKU: An evidence and consensus based approach. *Mol Genet Metab*2016;118(2):72–83.

2. van Wegberg AMJ, MacDonald A, Ahring K, et al. The complete European guidelines on phenylketonuria: Diagnosis and treatment. *Orphanet J Rare Dis*2017 Oct;12(1).

3. Inwood A, Lewis K, Balasubramaniam S, et al. Australasian consensus guidelines for the management of phenylketonuria (PKU) throughout the lifespan [Internet]. 2017. p. 1–36. Available from: https://www.mdda.org.au/resources/handbooks-guidelines/

4. Firman S, Witard OC, O'Keeffe M, Ramachandran R. Dietary protein and protein substitute requirements in adults with phenylketonuria: A review of the clinical guidelines. *Clin Nutr*2020 Nov 11. http://www.sciencedirect.com/science/article/pii/S0261561420306075 (cited 2020 Nov 17)

5. Yi SH, Singh RH. Protein substitute for children and adults with phenylketonuria. *Cochrane Database Syst Rev*2015;2015(2).

6. MacDonald A, van Wegberg AMJ, Ahring K, et al. PKU dietary handbook to accompany PKU guidelines. *Orphanet J Rare Dis*2020 Aug 4;15(1):171.

7. MacDonald A, Ashmore C, Daly A, et al. An Observational Study Evaluating the Introduction of a Prolonged-Release Protein Substitute to the Dietary Management of Children with Phenylketonuria. *Nutrients*2020 Sep 3 [cited 2021 May 17];12(9). Available from: https://www.ncbi.nlm.nih.gov/pmc/articles/PMC7551143/

8. Scheinin M, Barassi A, Junnila J, et al. Amino Acid Plasma Profiles from a Prolonged-Release Protein Substitute for Phenylketonuria: A Randomized, Single-Dose, Four-Way Crossover Trial in Healthy Volunteers. *Nutrients*2020 Jun 2 [cited 2021 May 17];12(6). Available from: https://www.ncbi.nlm.nih.gov/pmc/articles/PMC7352445/

9. Giovannini M, Riva E, Salvatici E, et al. Randomized controlled trial of a protein substitute with prolonged release on the protein status of children with phenylketonuria. *J Am Coll Nutr*2014;33(2):103–10.

10. Rand WM, Pellett PL, Young VR. Meta-analysis of nitrogen balance studies for estimating protein requirements in healthy adults. *Am J Clin Nutr*2003;77(1):109–27.

11. Rodriguez NR. Introduction to Protein Summit 2.0: Continued exploration of the impact of high-quality protein on optimal health. *Am J Clin Nutr*2015;101(6):1317S-1319S.

For peer review only - http://bmjopen.bmj.com/site/about/guidelines.xhtml

12. Rodriguez N, Miller S. Effective translation of current dietary guidance: Understanding and communicating the concepts of minimal and optimal levels of dietary protein. *Am J Clin Nutr*2015;101(6):1353S-1358S.

13. Gropper SS, Acosta PB. Effect of simultaneous ingestion of L-amino acids and whole protein on plasma amino acid and urea nitrogen concentrations in humans. *J Parenter Enter Nutr*1991;15(1):48–53.

14. Metges CC, El-Khoury AE, Selvaraj AB, et al. Kinetics of L-[1-13C]leucine when ingested with free amino acids, unlabeled or intrinsically labeled casein. *Am J Physiol Endocrinol Metab*2000;278(6 41-6):1000–9.

15. Dangin M, Boirie Y, Guillet C, et al. Influence of the protein digestion rate on protein turnover in young and elderly subjects. *J Nutr*2002;132(10):3228–33.

16. Dangin M, Boirie Y, Garcia-Rodenas C, et al. The digestion rate of protein is an independent regulating factor of postprandial protein retention. *Am J Physiol Endocrinol Metab*2001;280(2 43-2):340–8.

17. Boirie Y, Dangin M, Gachon P, et al. Slow and fast dietary proteins differently modulate postprandial protein accretion. *Proc Natl Acad Sci U S A*1997;94(26):14930–5.

18. Witard O, Wardle S, Macnaughton L, et al. Protein Considerations for Optimising Skeletal Muscle Mass in Healthy Young and Older Adults. *Nutrients*2016 Mar 23;8(4):181.

19. Paddon-Jones D, Campbell WW, Jacques PF, et al. Protein and healthy aging. *Am J Clin Nutr*2015;101(6):1339S-1345S.

20. Moore DR, Areta J, Coffey VG, et al. Daytime pattern of post-exercise protein intake affects whole-body protein turnover in resistance-trained males. *Nutr Metab*2012;9(1):91.

21. Liao CD, Tsauo JY, Wu YT, et al. Effects of protein supplementation combined with resistance exercise on body composition and physical function in older adults: a systematic review and meta-analysis. *Am J Clin Nutr*2017 Oct;106(4):1078–91.

22. Witard OC, Jackman SR, Breen L, et al. Myofibrillar muscle protein synthesis rates subsequent to a meal in response to increasing doses of whey protein at rest and after resistance exercise. *Am J Clin Nutr*2014 Jan;99(1):86–95.

23. Shad BJ, Thompson JL, Holwerda AM, et al. One Week of Step Reduction Lowers Myofibrillar Protein Synthesis Rates in Young Men. *Med Sci Sports Exerc*2019;51(10):2125–34.

24. Smith GI, Reeds DN, Hall AM, et al. Sexually dimorphic effect of aging on skeletal muscle protein synthesis. *Biol Sex Differ*2012 May 23;3(1):11.

For peer review only - http://bmjopen.bmj.com/site/about/guidelines.xhtml

25. Moore DR, Churchward-Venne TA, Witard O, et al. Protein ingestion to stimulate myofibrillar protein synthesis requires greater relative protein intakes in healthy older versus younger men. *J Gerontol A Biol Sci Med Sci*2015;70(1):57–62.

26. Cuthbertson D, Smith K, Babraj J, et al. Anabolic signaling deficits underlie amino acid resistance of wasting, aging muscle. *FASEB J*2005;19(3):1–22.

27. Burd NA, Tang JE, Moore DR, et al. Exercise training and protein metabolism: influences of contraction, protein intake, and sex-based differences. *J Appl Physiol Bethesda Md*1985. 2009 May;106(5):1692–701.

28. Doulgeraki A, Skarpalezou A, Theodosiadou A, et al. Body composition profile of young patients with phenylketonuria and mild hyperphenylalaninemia. *J Endocrinol Metab*2014 Jul;12(3):e16061.

29. Evans M, Truby H, Boneh A. The relationship between dietary intake, growth and body composition in Phenylketonuria. *Mol Genet Metab*2017;122(1–2):36–42.

30. Mazzola PN, Nalin T, Castro K, et al. Analysis of body composition and nutritional status in Brazilian phenylketonuria patients. *Mol Genet Metab Rep*2016 Mar;1:16–20.

31. Huemer M, Huemer C, Moslinger D, et al. Growth and body composition in children with classical phenylketonuria: results in 34 patients and review of the literature. *J Inherit Metab Dis*2007 Oct;30(5):694–9.

32. Sailer M, Elizondo G, Martin J, et al. Nutrient intake, body composition, and blood phenylalanine control in children with phenylketonuria compared to healthy controls. *Mol Genet Metab Rep*2020;23.

33. Rocha JC, van Spronsen FJ, Almeida MF, et al. Early dietary treated patients with phenylketonuria can achieve normal growth and body composition. *Mol Genet Metab*2013;1:S40-43.

34. Rocha JC, van Spronsen FJ, Almeida MF, et al. Dietary treatment in phenylketonuria does not lead to increased risk of obesity or metabolic syndrome. *Mol Genet Metab*2012 Dec;107(4):659–63.

35. PROSPERO: International prospective register of systematic reviews. Search term: Phenylketonuria. Available from: https://www.crd.york.ac.uk/prospero/#searchadvanced (accessed 01 Feb 2021)

36. Arksey H, O'Malley L. Scoping studies: towards a methodological framework. *Int J Soc Res Methodol*2005 Feb;8(1):19–32.

37. Levac D, Colquhoun H, O'Brien KK. Scoping studies: advancing the methodology. *Implement Sci*2010 Dec;5(1):69.

For peer review only - http://bmjopen.bmj.com/site/about/guidelines.xhtml

38. Pawliuk C, Brown HL, Widger K, et al. Optimising the process for conducting scoping reviews. *BMJ Evid-Based Med*2020 Oct 21;bmjebm-2020-111452. doi: 10.1136/bmjebm-2020-111452.

39. Peters MDJ, Godfrey C, McInerney P, Munn Z, Tricco AC, Khalil, H. Chapter 11: Scoping Reviews (2020 version). In: Aromataris E, Munn Z (Editors). JBI Manual for Evidence Synthesis, JBI, 2020. Available from https://synthesismanual.jbi.global. https://doi.org/10.46658/JBIMES-20-12

40. Tricco AC, Lillie E, Zarin W, et al. PRISMA Extension for Scoping Reviews (PRISMA-ScR): Checklist and Explanation. *Ann Intern Med*2018 Sep 4;169(7):467–73.

For peer review only

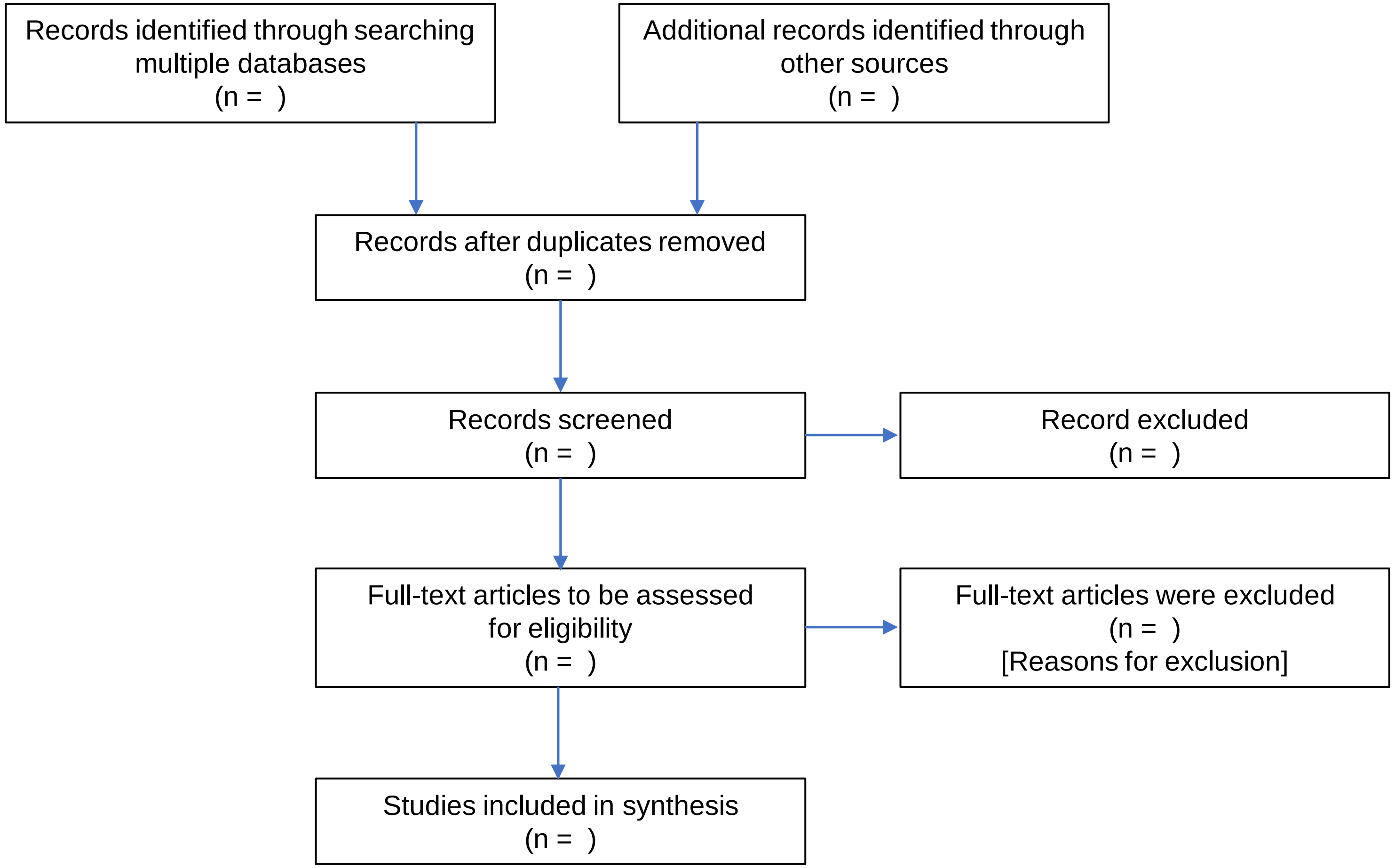

Figure 1: Preferred Reporting Items for Systematic Reviews and Meta-Analysis flow diagram for scoping review process. Retrieved from Tricco et al.

Supplemental material: Search strategy

Database: MEDLINE (Ovid) < 1946 to May 17, 2021>

Search	Query	Records retrieved
#1	exp Phenylketonurias/ OR Phenylketonurias.mp. OR PKU.mp. OR Hyperphenylalanin?emia.mp. OR exp Phenylalanine Hydroxylase/ OR "Phenylalanine Hydroxylase".mp. OR "Phenylalanine Hydroxylase deficiency".mp.	8935
#2	exp Nutritional Status/ OR "nutritional status".mp. OR "protein status".mp. OR exp Muscle Proteins/ OR "muscle proteins".mp. OR "protein metabolism".mp. OR "muscle protein metabolism".mp. OR exp Body Composition/ OR "body composition".mp. OR exp Muscle Strength/ OR "muscle strength".mp. OR "muscle function".mp. OR exp Prealbumin/ OR prealbumin.mp. OR Transthyretin.mp. OR exp Albumins/ OR albumin.mp. OR "3-methylhistidine concentrations".mp. OR exp Retinol-Binding Proteins/ OR "retinol-binding protein".mp. OR "urea production".mp. OR exp Nitrogen/ OR nitrogen.mp. OR exp Creatinine/ OR creatinine.mp. OR “VO2max” OR exp Physical Exertion/ OR "physical exertion".mp. OR exp Exercise Test/ OR "exercise test".mp.	1093416
#3	#1 AND #2	**282**
Limit	English only	**255**

For peer review only - http://bmjopen.bmj.com/site/about/guidelines.xhtml
